# Echocardiographic assessment and new advances of right ventricle function in rats with pulmonary hypertension

**DOI:** 10.3389/fphar.2025.1682105

**Published:** 2025-11-27

**Authors:** Weijie Wang, Ming Ren, Dianxiang Lu

**Affiliations:** 1 Department of Cardiovascular Disease, The Affiliated Hospital of Qinghai University, Xining, China; 2 Clinical Medical College, Affiliated Hospital of Chengdu University, Chengdu, China

**Keywords:** ultrasound imaging, pulmonary hypertension, rat model, right heart function, multimodal fusion

## Abstract

Pulmonary Hypertension (PH) is a pathophysiological disease characterized by abnormally elevated pulmonary artery pressures due to a variety of known or unknown causes, which may ultimately lead to right ventricle (RV) failure or even death. The rat has become a major animal model of PH study because of its low cost, genetic control, and similarity of pathologic features to humans. The assessment of right heart function is crucial for basic research on PH and the diagnosis/treatment of PH diseases. Ultrasound imaging has become one of the main methods for assessing right heart function because of its noninvasive measurement, its **ability** to dynamically monitor the morphology and function of the rat heart, and its low cost and ease of use. Moreover, the new ultrasound technology of speckle tracking is able to detect myocardial dysfunction earlier and more sensitively, and three-dimensional ultrasound generates three-dimensional images of the heart, which is able to more accurately assess the morphology and functional changes of the right heart. In addition, correlation analysis of ultrasound indexes with the gold standard found that pulmonary acceleration time (PAT), PAT/pulmonary ejection time (PET) measured by ultrasound with pulmonary artery systolic pressure (PASP) measured by right heart catheterization (RHC); PAT measured by ultrasound with mean pulmonary artery pressure (mPAP) by RHC; and right ventricular free wall thickness (RVFWT) measured by ultrasound with Fulton’s index, showed good correlation. In this paper, we reviewed the pathologic changes of the heart in PH, the modeling methods of PH rat model, ultrasound imaging for the assessment of right heart function and the advantages of speckle tracking and three-dimensional ultrasound as emerging ultrasound technologies. And we also proposed that the multimodal fusion of ultrasound and cardiac magnetic resonance (CMR), micro computed tomography (micro-CT), artificial intelligence (AI) could be the future direction of cardiac function assessment in PH rat. Accurate assessment of right ventricular function is crucial for further research into the molecular mechanisms of PH and drug screening.

## Highlights

- The symptoms of pulmonary hypertension are severe, and **RV failure** is the outcome. In patients with PH, right ventricular function status is the most critical independent prognostic predictor. Animal models can more accurately simulate the pathophysiological characteristics and therapeutic responses of human diseases. Therefore, assessment of right heart function is crucial. Ultrasound imaging technology has become a very important tool for assessing right heart function in rats because of its **ability** to provide *in vivo*, non-invasive, dynamic assessment.
- Correlation analysis of ultrasound indexes with the gold standard was done and found that PAT, PAT/PET measured by ultrasound with PASP measured by RHC; PAT measured by ultrasound with mPAP by RHC; and RVFWT measured by ultrasound with Fulton’s index, showed good correlation.
- The multimodal fusion of ultrasound and MRI or micro-CT or AI might be the future development trend and direction of cardiac function assessment in PH rat.
- Accurate assessment of right ventricular function in rats with pulmonary hypertension is crucial in basic research on the molecular mechanisms and drug screening of PH.

## 1 Introduction

Pulmonary hypertension (PH) is a pathophysiological disease characterized by abnormally elevated pulmonary arterial pressures due to a variety of known or unknown causes, which may eventually lead to RV failure or even death. Its diagnostic criterion is a mean pulmonary artery pressure (mPAP) ≥20 mmHg (≥25 mmHg in the old criterion) measured by right heart catheterization (RHC) at sea level and at rest. The etiology of PH is complex and varied, the World Health Organization has classified PH into five clinical subgroups: pulmonary arterial hypertension (PAH) (group 1); PH due to left-sided heart disease (group 2); PH due to lung disease and/or hypoxemia (group 3); PH due to pulmonary artery obstruction including chronic thromboembolism (group 4), and PH related to multifactorial causes (group 5) (Johnson et al., 2023). The morbidity and mortality of PH are high and have been on the rise in recent years. Currently, PH affects at least 1% of the world’s population and is more prevalent in low- and middle-income countries (Mocumbi et al., 2024; Hoeper et al., 2016). PH due to left-sided heart disease, PH due to lung disease and/or hypoxemia is the most common type in the clinic. Patients with PH often present clinically with symptoms such as dyspnea, fatigue, and chest pain, and with the disease progressing, it may lead to RV failure or even death (Johnson et al., 2023). Therefore, the assessment of right heart function is crucial in basic research and clinical diagnosis and treatment of PH.

The rat has become a major animal model for basic PH research because of its low cost, genetic control, and similarity of pathological features to humans (Maarman et al., 2013; Sztuka and Jasińska-Stroschein, 2017). Firstly, the feeding and experimental equipment cost of rats is relatively low. Secondly, the establishment methods of rat model are relatively simple, such as chronic hypoxia induction method and monocrotaline (MCT) induction method, *etc.*, and the cost of reagents and operation is also low. In addition, the genetic background of rats is relatively clear, which can reduce the influence of genetic heterogeneity on the experimental results and improve the reliability and reproducibility of the study. More importantly, the rat PH models can mimic the key pathological features of human PH, such as endothelial cell injury, smooth muscle cell proliferation, vascular remodeling and right heart hypertrophy (Wu et al., 2022; Boucherat et al., 2022). For example, the rat PH model induced by MCT is more similar in pathophysiological changes to the PH caused by inflammation in humans (Maarman et al., 2013); the rat PH model induced by chronic hypoxic exposure can lead to pulmonary vasoconstriction, vascular remodeling, and right cardiac hypertrophy, which is also consistent with the pathophysiological process of chronic hypoxic PH in humans (Ryan et al., 2011; Cai et al., 2023). Moreover, the response of PH therapeutic drugs in rats are also similar to those in humans, and can be used for drug screening and efficacy assessment to provide the basis for clinical trials (Dignam et al., 2022).

Among the cardiac function assessment tools for rats with PH, ultrasound imaging is an *in vivo*, noninvasive and dynamically monitorable examination method, which allows real-time observation of structural and functional changes in the heart, providing detailed information about the changes of the heart in different physiologic and pathologic states. For example, during the establishment of the PH rat model, ultrasound imaging can be performed periodically to assess the changes in right ventricular function, thus providing a better understanding of the disease process and the effects of drug therapy (Thijssen and de Korte, 2014; Sztechman et al., 2020). Moreover, the emerging speckle tracking technology in ultrasound imaging can analyze the trajectory and strain of the myocardium by tracking the echo speckle signals in the myocardial tissues, and sensitively detect abnormalities in myocardial function, providing a basis for the assessment and early diagnosis of the right cardiac function in PH (Mondillo et al., 2011). In addition, three-dimensional (3D) ultrasound technology in ultrasound imaging can more intuitively and comprehensively display the morphology and volume of right ventricular (RV), *etc.*, which assess the structure and function of the heart more comprehensively and accurately (La et al., 2018). Futhermore, 3D ultrasound can be combined with other technology such as speckle tracking, cardiac magnetic resonance (CMR), micro computed tomography (micro-CT), and artificial intelligence (AI) to further improve the assessment of right ventricular function in terms of accuracy and precision (Gillam and Marcoff, 2024).

**FIGURE 1:**
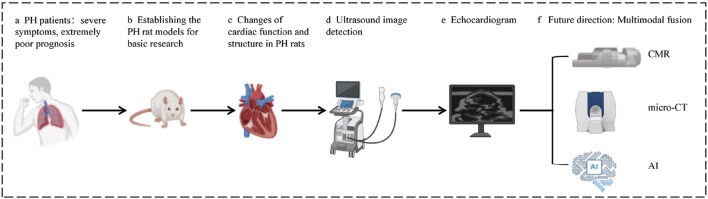
**(a)** PH patients: severe symptoms, extremely poor prognosis. **(b)** Establishing the PH rat models for basic mechanism research on PH. **(c)** Changes of cardiac function and structure in PH rats. **(d)** Ultrasound image detection. **(e)** Echocardiogram. **(f)** Future direction: multimodal integration. Original figure created with BioRender.com. PH, Pulmonary hypertension; CMR, Cardiac magnetic resonance; micro-CT, Micro computed tomography; AI, Artificial intelligence.

In this paper, we review the pathologic changes of the heart in PH, the application of ultrasound imaging in the assessment of the right heart in PH rat, the advantages of emerging ultrasound technologies (speckle tracking and three-dimensional ultrasound imaging), and the future direction of cardiac function assessment. We hope that it will provide an important reference for the research on molecular mechanisms and drug screening of PH. Figure 1 provides an overview of the content included in this review.

## 2 Pathologic changes of the heart in PH

PH leads to pathological changes such as right ventricular hypertrophy (RVH) and RV failure. RV failure is caused by the combination of pathological inflammation (Hassoun, 2021), endothelial dysfunction (Park et al., 2021), vascular remodeling (Ichimura et al., 2024), right ventricular remodeling (Park et al., 2021), metabolic disorders (Fowler et al., 2019), myocardial fibrosis (Vang et al., 2021), atrial dilatation (Wessels et al., 2022), disturbances in autonomic regulation and genetic factors (Wessels et al., 2023a; Vonk et al., 2019).

**FIGURE 2:**
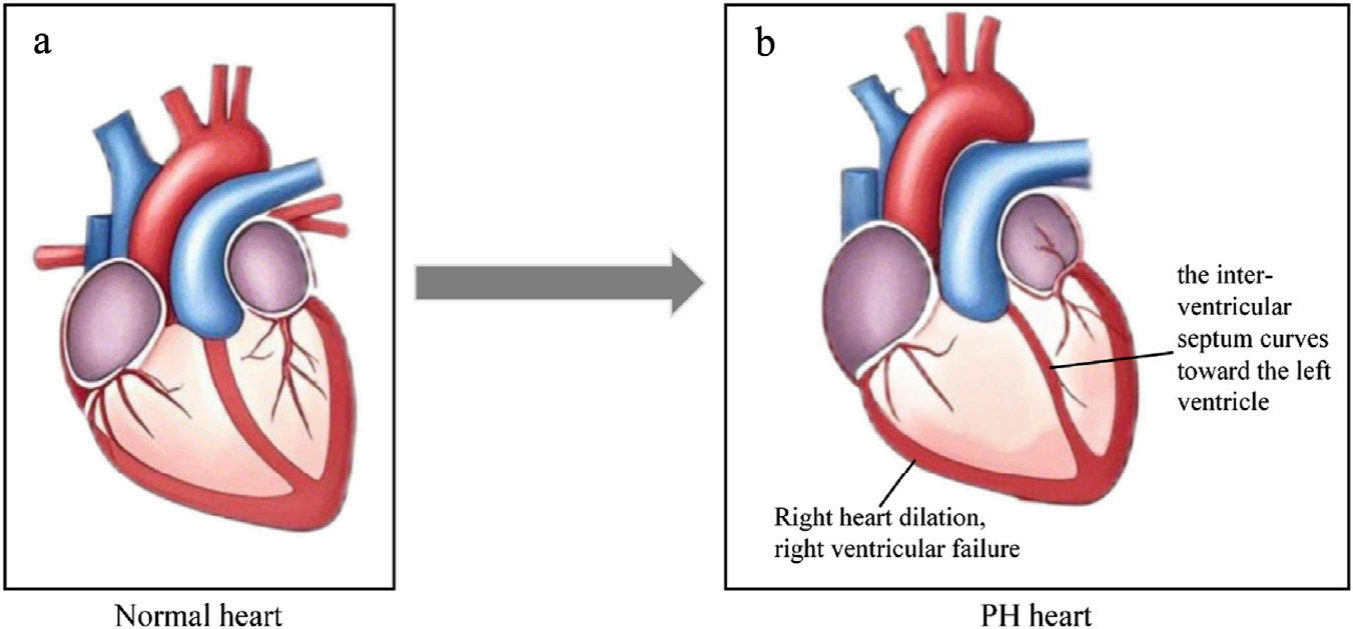
Cardiologic changes in PH. **(a)** Normal heart. **(b)** The heart in PH. The right heart dilation, right ventricular failure, the interventricular septum curves toward the left ventricle, and RV-PA become uncoupled. Original figure created with BioRender.com. PH, Pulmonary hypertension; RV-PA, Right ventricle and pulmonary artery.

In the early stage of PH, elevated pulmonary artery pressure increases the afterload on RV, which in turn leads to progressive hypertrophy of RV to enhance myocardial contractility and maintain stable cardiac output (CO) (Vonk-Noordegraaf et al., 2013; Llucià-Valldeperas et al., 2021; Zelt et al., 2022). In the late stages of PH, long-term elevated pulmonary artery pressure causes a sustained increase in right ventricular afterload, which in turn leads to right heart dilatation, myocardial dysfunction and RV failure (Wessels et al., 2022; Vonk Noordegraaf et al., 2017; Wessels et al., 2023b). Firstly, long-lasting pressure overload of RV causes myocardial metabolic disorders, which in turn lead to myocardial dysfunction (Chan and Rubin, 2017; Agrawal et al., 2022; Kazmirczak et al., 2023). Besides, long-term elevated pulmonary artery pressure also leads to thickening of the right ventricular wall and reduced perfusion of the coronary arteries within the myocardium, which further exacerbate myocardial ischemia, hypoxia and myocardial dysfunction. In addation, chronic pressure overload leads to further progressive deterioration of right ventricular function, ultimately leading to right ventricular failure (Hassoun, 2021; Tello et al., 2019). Moreover, pathologic changes in the RV may result in the curvature of the interventricular septum toward the left ventricle, left ventricular diastolic restriction, and decreased cardiac output (Kishiki et al., 2019; Hardegree et al., 2013; Bousseau et al., 2023). Figure 2 demonstrates the pathologic changes of the heart in PH.

## 3 Commonly used PH rat models and their model characteristics

The main commonly used rat models of PH include the MCT-induced PH rat model, the chronic hypoxia-induced PH rat model, the Sugen5416/hypoxia (SuHx)-induced PH rat model, and the bypass surgery-induced PH rat model. Table 1 summarizes the characteristics of above commonly used rat models of PH, including modeling methods, advantages, limitations, and types of human PH that can be simulated.

**TABLE 1:** Characteristics of currently used rat PH models.

Model types	Establishment methods	Advantages	Limitations	Key hemodynamic changes	The main echo cardiographic features	Best applications
The MCT- induced PH rat model	Rat is given a single subcutaneous/peritoneal injection of MCT (60–80 mg/kg). The model development timeline is 3–4 weeks.	Easy to perform, reproducible, low cost, mPAP elevation, PVR elevation, RVH, thickening of the intima-media of the small pulmonary arteries, mainly for inflammation-related PAH studies.	No severe vaso-occlusive lesions (tufted lesions and intimal hyperplasia) as humans; MCT is more toxic and may have some toxic effects on other organs such as the liver and kidneys, in addition to damage to the pulmonary vasculature.	mPAP, PVR, RVSP elevated.	RVFWT increased; TAPSE markedly decreased; PAT/PET decreased.	Mimicking inflammation-related PAH (group 1)
The chronic hypoxia-induced PH rat model	Rat is exposed to hypoxia (typically 10% O2). The model development timeline is 3–4 weeks.	Good reproducibility; mainly used to study the PH caused by hypoxia.	Relatively mild degree of induced PH, insignificant vascular remodeling, no severe vaso-occlusive lesions (tufted lesions and intimal hyperplasia); only mimicing mild/moderate PH.	mPAP, RVSP elevated.	RVFWT mildly increased; TAPSE, PAT/PET mildly decreased.	Mimicking PH caused by lung disease and/or hypoxemia (group 3)
The SuHx- induced PH rat model	Rat is given a single subcutaneous injection of Sugen5416 (20 mg/kg), then exposed to hypoxia (generally 10% O2) for 3–4 weeks, and then restored to normoxia for 3–5 weeks.	The method can be induced to severe PAH with clumping like lesions and right ventricular failure; this model does not reverse when placed in normoxia; lesion organs are mainly confined to the lungs.	No perivascular infiltration of inflammatory cells (macrophages/monocytes); large differences in PH models between strains (e.g., Fischer and SD rats).	mPAP, RVSP elevated PVR elevated significantly.	RVFWT significantly increased; TAPSE significantly decreased; PAT/PET decreased.	Mimicking PAH (group 1)
The shunt surgery-induced PH rat model	Rat is operated to establish abnormal aorta-pulmonary artery or aorta-inferior vena cava blood flow pathway. The model development timeline is 3–4 weeks.	Mimicing pathologic changes in CHD-PAH.	Complicated surgical operation and difficult modeling techniques; higher postoperative mortality; high surgical risk and more complications.	mPAP, RVSP significantly elevated.	RVFWT increased; TAPSE and PAT/PET decreased.	Mimicking CHD-PAH (group 1)
The pulmonary arterial banding-induced rat model	A thoracotomy was performed on rats to isolate the pulmonary artery. A needle was inserted along the pulmonary artery, inducing a fixed contraction proportional to the needle gauge. The modeling period lasts approximately 5 weeks.	Right ventricular remodeling and dysfunction without affecting pulmonary vasculature.	The procedure is relatively complex and requires advanced technical skills It can serve as a model for right heart failure but does not induce pulmonary vascular remodeling or pulmonary arterial hypertension Postoperative mortality rates in animals are relatively high.	mPAP, RVSP elevated.	RVFWT increased; TAPSE and PAT/PET significantly decreased.	Mimicking right heart failure (late-stage PAH symptoms)
The high-fat diet (HFD) + SU5416 rat model	A high-fat diet administered for 8–12 weeks induces metabolic syndrome, followed by a single subcutaneous injection of SU5416 (20 mg/kg). The high-fat diet is continued for 4–6 weeks post-injection.	Clinical characteristics of PH-HFpEF associated with simulated metabolic syndrome (obesity, insulin resistance); non-invasive, simple to operate.	Long modeling period (≥8 weeks); Mild PH severity (limited increase in mPAP); Weak pulmonary vascular remodeling.	RVSP mildly elevated; LVEDP elevated.	RVFWT mildly increased; TAPSE, PAT/PET decreased.	Mimicking early Mechanisms of PH-HFpEF Associated with Metabolic Syndrome
The ZSF1 obese rats + SU5416 model	Directly using genetically obese ZSF1 rats (leptin receptor-deficient), administer a single subcutaneous injection of SU5416 (20–100 mg/kg) for 3–6 weeks.	Spontaneously exhibits HFpEF phenotype (diastolic dysfunction, exercise intolerance); stable recurrence of CpcPH (mixed pulmonary hypertension).	Reliance on specialized genetic strains results in high costs; SU5416 dosage requires optimization (high doses may cause right heart failure).	RVSP significantly increased; mPAP and PVR markedly elevated.	RVFWT significantly increased; TAPSE and PAT/PET decreased.	Mimicking critical illness CPCPH (especially with HFpEF)

Abbreviations: MCT, monocrotaline; SuHx, Sugen5416/hypoxia; Sugen5416, SU5416; High-fat diet, HFD; PH, pulmonary hypertension; PAH, pulmonary arterial hypertension; mPAP, mean pulmonary artery pressure; PVR, pulmonary vascular resistance; CHD-PAH, Congenital heart disease-pulmonary arterial hypertension; RVH, right ventricular hypertrophy; RVSP, right ventricular systolic pressure; PH-HFpEF, pulmonary hypertension due to heart failure with preserved ejection fraction; CpcPH, Combined pre- and capillary Pulmonary Hypertension; LVEDP, Left Ventricular End-Diastolic Pressure.

The MCT-induced PH rat model is established by a single intraperitoneal or subcutaneous injection of MCT (60–80 mg/kg). Toxic metabolites produced by MCT *in vivo* can directly damage pulmonary vascular endothelial cells, leading to endothelial cell dysfunction and apoptosis, which triggers pulmonary vasoconstriction and vascular remodeling, increases pulmonary vascular resistance, and ultimately developing PH (Maarman et al., 2013). In this model, the disease progresses rapidly. Obvious PH symptoms such as elevated mean pulmonary artery pressure (mPAP), elevated pulmonary vascular resistance (PVR), elevated right ventricular systolic blood pressure, and right cardiac hypertrophy can be seen within 3–4 weeks after MCT injection. As the disease progressing, this model develops right ventricular dysfunction and RV failure (Morimatsu et al., 2012). In addition, this model has obvious pulmonary vascular pathologic changes, which can be seen as pulmonary artery endothelial cell damage and thickening of the intima-media of the small pulmonary arteries, *etc.*, which is especially capable of mimicing the PH caused by inflammation in human PH (group 1) (Wu et al., 2022). Besides, this model is stable, and the physiological indexes and pathological changes are more consistent between different batches of rats under the same MCT dose and experimental conditions (Maarman et al., 2013). Although the model is simple to operate and the disease progresses quickly, MCT is more toxic and may cause some toxic effects on other organs such as liver and kidney in addition to the damage to the pulmonary vasculature. In addition, its PH pathology is not fully consistent with human PH and lacks some of the characteristic changes in human PH, such as the absence of severe vaso-occlusive lesions (tufted lesions and endothelial hyperplasia) (Boucherat et al., 2022).

The chronic hypoxia-induced PH rat model was established by exposing rats to the hypoxic environment, mimicing the hypoxic conditions of certain pathological states or plateau environments, setting the oxygen concentration at about 10% (equivalent to about 7,000 m above sea level), and continuing the exposure for a certain period of time (3–4 weeks) (Irwin et al., 2015), which resulted in pulmonary vasoconstriction, elevated pulmonary artery pressure, as well as pulmonary vascular endothelial cell dysfunction, smooth muscle cell proliferation and vascular remodeling, and ultimately the development of PH (Ma et al., 2015). With prolonged hypoxic exposure, right ventricular hypertrophy occurs, but right heart dysfunction occurs relatively late (Cai et al., 2023). The model has a narrow scope of application and is mainly used to study PH caused by lung diseases and/or hypoxemia (group 3), and is not comprehensive enough to mimic other types of PH in humans. The model is relatively simple to operate, only need to control the hypoxic environment, no need to use special drugs, but the hypoxia chamber equipment is expensive. In addition, this model induces a relatively mild degree of PH, insignificant vascular remodeling, and slow disease progression for a long experimental period.

The Sugen/hypoxia (SuHx)-induced PH rat model was established by subcutaneously injecting rats with Sugen5416 (typically 20 mg/kg), and exposing them to the hypoxic environment (typically 10% O_2_) for a certain period of time (typically 3–4 weeks), and then restoring normoxia (typically 3–5 weeks). Sugen5416 is an inhibitor of vascular endothelial growth factor receptor-2 (VEGFR-2). Under hypoxic conditions, Sugen5416 leads to apoptosis of endothelial cells while stimulating the proliferation of new endothelial cells and smooth muscle cells, which causes pathological remodeling of the pulmonary vasculature, and meanwhile, leads to increased pulmonary arterial pressures and right ventricular remodeling as well as severe RV failure. Under the combined effect of hypoxia and Sugen5416, the formation and development of PH in this model has sereval certain stage. A gradual evolution from early pulmonary vasoconstriction to late vascular remodeling and right ventricular dysfunction can be observed, and even severe PH could be induced ultimately (Boucherat et al., 2022; Bikou and Sassi, 2024). This model not only has pulmonary vascular remodeling and right ventricular remodeling, but also has plexiform lesions and right ventricular failure. And the pathological features of this model cannot be reversed after being placed in normoxia, as well as the diseased organs are mainly targeted to the lungs, which is able to better mimic the pathogenesis of human PH (group 1), and is suitable for long-term and in-depth mechanistic studies of human PH (group 1). However, this model has some limitations, such as the fact that human inflammatory cell (macrophage/monocyte) infiltration is usually not seen in its perivascular periphery, and there is a large variation between strains [e.g., Fischer rats and Sprague Dawley (SD) rats] (Ryan et al., 2011; Suen et al., 2019).

The shunt surgery-induced PH rat model is established by creating abnormal aorta-pulmonary artery or abdominal aorta-inferior vena cava blood flow pathway for typically 3–4 weeks (Meng et al., 2013; Garcia and Diebold, 1990). The abnormal blood flow channels lead to increased blood flow in the pulmonary circulation and elevated pulmonary artery pressure. Long-term elevated pulmonary artery pressure causes pulmonary vascular remodeling, significant increase in right ventricular hypertrophy index, right ventricular dysfunction, *etc.* These pathological changes are similar to PH caused by congenital heart disease (CHD) in humans, and better mimic the PH caused by CHD in human PH (group 1) (Stenmark et al., 2009; Bisserier et al., 2022). However, this model requires microsurgical techniques for vascular anastomosis, which needs a high level of experimenter skill. In addition, there are many postoperative complications, high surgical mortality (up to 10% within 24 h after surgery) and poor consistency of pathological manifestations in surviving animals. In addition, the degree of pulmonary artery pressure elevation is affected by the fractional flow, which makes the experiment less controllable (Meng et al., 2013; Garcia and Diebold, 1990).

The PAB model involves placing an adjustable band on the main pulmonary artery of rats to create a fixed stenosis, thereby increasing resistance to right ventricular ejection (Maarman et al., 2013). The band creates fixed outflow tract obstruction, causing a sharp rise in right ventricular systolic pressure (RVSP) and elevated pulmonary artery pressure (PAP). Over time, the right ventricle undergoes a progression from compensation to decompensation, developing compensatory hypertrophy that eventually evolves into RV failure. The primary pathological changes occur in the right ventricle, with no pulmonary vascular pathology (Wang et al., 2017; Shanks et al., 2009; Vandamme, 2015). It is commonly used to simulate right ventricular pressure overload diseases (group 2), such as congenital right ventricular outflow tract obstruction caused by pulmonary valve stenosis. It is frequently employed to study right ventricular remodeling under pressure overload and the molecular mechanisms of RV failure (Wang et al., 2017; Stenmark et al., 2009; Hayashi et al., 2025). Additionally, this model offers controllable manipulation and excellent reproducibility. However, the procedure is technically demanding, requiring skilled microsurgical techniques, and carries a relatively high operative mortality rate (Wang et al., 2017; Shanks et al., 2009; Vandamme, 2015).

## 4 Pharmacological studies in a rat model of PH

Pharmacological research in rat models of PH primarily focuses on exploring novel compounds, drug delivery systems, and signaling pathway regulatory mechanisms.

For instance, Dhananjayan et al. (2025) found that the EPA derivative 5,6-diHETE lactone improves MCT-induced rat PH by activating the endothelial PLC-IP3 signaling pathway, thereby reducing mean pulmonary artery pressure and pulmonary vascular remodeling. Italian researchers (Thomas et al., 2009) reported that the novel compound sotatercept alleviates pulmonary vascular remodeling in the MCT model by inhibiting SMAD2 phosphorylation, among other mechanisms.

Additionally, Chida-Nagai et al. (2025) developed pulmonary artery-targeted metformin nanocapsules that significantly improved hemodynamics and right ventricular hypertrophy in MCT rats by activating the AMPK pathway.

Furthermore, multiple studies including those by Sanada et al. (2021), Yung et al. (2016) confirmed activation of the TGF-β/SMAD pathway in PH models. Targeted intervention was shown to inhibit pulmonary vascular smooth muscle proliferation, thereby exerting therapeutic effects against PH.

## 5 Ultrasonographic detection in PH rat models

Among the cardiac function assessment tools for rats with PH, ultrasound imaging is an *in vivo*, noninvasive and dynamically monitorable examination method. The basic imaging modalities of ultrasound imaging are two-dimensional (2D), M-mode, pulsed wave Doppler (PWD), and color Doppler, and tissue Doppler imaging (Hockstein et al., 2021).

Two-dimensional imaging, the most basic imaging modality, provides real-time two-dimensional images of the heart and can be used to measure the morphology, size of the heart, such as ventricular internal diameter, the ventricular wall thickness, etc (Brugger et al., 2021). M-mode is used to acquire one-dimensional image of the linear region of interest over time by selecting the region on two-dimensional image, such as tricuspid annular plane systolic excursion (TAPSE), fractional shortening (FS), *etc.* The color Doppler mode visualizes the direction of blood flow and helps to detect blood flow abnormalities such as reflux. Pulse Doppler mode is used to analyze blood flow velocity and assess blood flow in a vessel of interest. The sampling volume is placed inside the vessel to measure parameters such as peak tricuspid flow velocity of the early rapid filling wave (E), peak velocity of the late filling wave due to atrial contraction (A). Tissue Doppler imaging (TDI) is different from pulse Doppler. TDI is mainly used to measure the motion velocity of myocardial tissue and can be used to measure myocardial systolic and diastolic function indexes, such as tissue Doppler-derived right ventricular systolic excursion velocity (S′), early diastolic myocardial relaxation velocity (E′) (Kjærgaard, 2012; Smolarek et al., 2017).

### 5.1 Commonly used indexes for ultrasound detection in PH rat models

**FIGURE 3:**
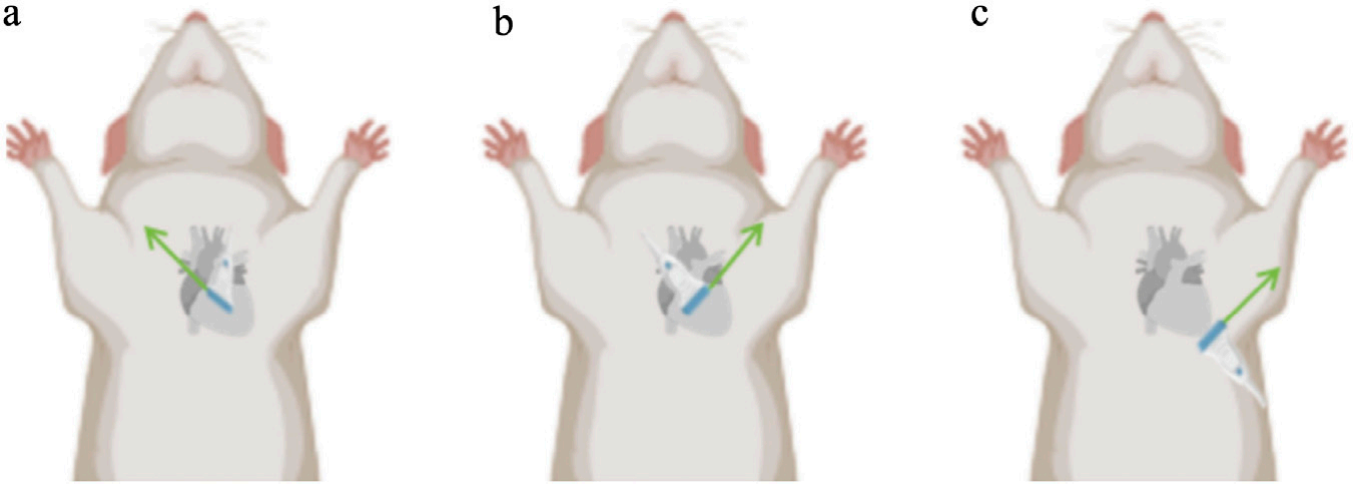
Ultrasound probe localization for cardiac ultrasound detection in PH rat. **(a)** Probe localization in the PLAX view, arrow indicates the direction of the probe marker. **(b)** Probe localization in the PSAX view, arrow indicates the direction of the probe marker. **(c)** Probe localization in the A4C view, arrow indicates the direction of the probe marker. Original figure created with BioRender.com. PH, Pulmonary hypertension; PLAX view, Parasternal long-axis view; PSAX view, Parasternal short-axis view; A4C view, Apical four-chamber view.

The indexes commonly used in the evaluation of the right heart in PH rats are: tricuspid annular plane systolic excursion (TAPSE), right ventricular systolic excursion velocity (S′), right ventricular fractional area change (RVFAC), E/E′, right ventricular free wall thickness (RVFWT), pulmonary acceleration time (PAT)/pulmonary ejection time (PET), TAPSE/PAT. Table 2 summarizes the characteristics of these commonly used indexes in PH rat models, including ultrasound measurement methods, the reference thresholds in PH rat models, significance, advantages, and limitations. The ultrasound windows commonly used to assess cardiac structure and function are the parasternal long-axis (PLAX) view, the parasternal short-axis (PSAX) view, and the apical four-chamber (A4C) view. Figure 3 demonstrates the probe positioning and the direction of the probe landmarks for these ultrasound windows.

**TABLE 2:** Commonly used indexes for ultrasound detection in PH rat.

Indexes	Measurement methods	Thresholds in PH rat	Significances	Advantages	limitations
TAPSE	In the A4C view, measuring the tricuspid annular plane systolic excursion from end-diastole to end-systole using M- mode in the tricuspid valve	—	Reflecting right ventricular systolic function, decreased TAPSE value suggests right ventricular systolic dysfunction	Simple measurement, less dependence on image quality, good reproducibility, good correlation with RVEF	Angle dependent measurement
S’	In the A4C view, measuring right ventricular systolic excursion peak velocity using tissue Doppler imaging in the tricuspid valve	—	Reflecting right ventricular systolic function, decreased S′ value suggests right ventricular systolic dysfunction	Repeatable, fast and easy measurement, reflecting the local contractile function of RV	Angle dependent measurement; does not reflect overall right ventricular function
RVFAC	In the A4C view, tracing the right ventricular endocardial border at end-diastole and end-systole, respectively, and calculating RVFAC [RVFAC = (end-diastolic area - end-systolic area)÷end-diastolic area×100%]	—	Reflecting overall right ventricular systolic function, decreased RVFAC value suggests right ventricular systolic dysfunction	Better reflection of overall right ventricular systolic function, good correlation with RVEF	Requiring accurate outlining of the endocardial border of RV; the area of the RVOT is ignored in measurements
E/E′	In the A4C view, measuring the peak tricuspid flow velocity of the early rapid filling wave using pulsed Doppler imaging and the early diastolic myocardial relaxation velocity using tissue Doppler imaging in the tricuspid valve, then calculating the E/E′ value	—	Reflecting right ventricular diastolic function, elevated E/E′ suggests increased right ventricular filling pressures	Indirect estimation of right ventricular pressure, valuable in the assessment of right heart diastolic function	Influenced by respiration and heart rate, does not fully reflect right ventricular diastolic function
RVFWT	In the A4C view, measuring the right ventricular free wall thickness in diastole using M-mode or 2D imaging	RVFWT ≥1.03 mm as the diagnostic threshold for RVH in rat	Reflecting the degree of hypertrophy of the right ventricular wall	Simple to use, easy to measure, used to assess RVH	Influenced by the morphology of RV and the position of the probe, needs to recognize accurately the endocardial edge of RV, does not fully reflect the overall structural changes in RV
PAT/PET	In the PSAX view, measuring pulmonary acceleration time and pulmonary ejection time in systole using pulsed Doppler in the pulmonary valve, then calculating the PAT/PET value	PAT/PET ≤0.25 as a diagnostic threshold for PH in rat	Reflecting pulmonary artery pressure, elevated PAT/PET suggests elevated pulmonary artery pressure	Valuable in assessing pulmonary artery pressure	The Doppler cursor needs to be aligned with the direction of blood flow during measurements, its accuracy is influenced by a number of factors including heart rate, right ventricular function, pulmonary artery compliance, and measurement technique

Abbreviations: TAPSE, Tricuspid annular plane systolic excursion; S’, Tissue Doppler-derived right ventricular systolic excursion velocity; RVFAC, Right ventricular fractional area change; E, Peak tricuspid flow velocity of the early rapid filling wave; E’, Early diastolic myocardial relaxation velocity; PAT, Pulmonary acceleration time; PET, Pulmonary ejection time; RVFWT, Right ventricular free wall thickness; PH, Pulmonary hypertension; RVH, Right heart hypertrophy; RVEF, Right ventricular ejection fraction; PLAX view, Parasternal long-axis view; PSAX view, Parasternal short-axis view; A4C view, Apical four-chamber view; RVOT, Right ventricular outflow tract; RV, Right ventricle; —, None.

#### 5.1.1 Tricuspid annular plane systolic excursion (TAPSE)

The TAPSE index is the measure of the distance that the right ventricular tricuspid annulus moves along the longitudinal plane during systole in the A4C view. It represents right ventricular longitudinal systolic function. During measurement, the M sampling wire is usually placed at the tricuspid annulus, and the longitudinal displacement of the annulus at peak contraction is measured to acquire TAPSE. This method is similar to the measurement of systolic displacement of the mitral annulus using TDI. The greater the amplitude of basal segment motion during systole, the greater the TAPSE value, which represents better ventricular systolic function (Aloia et al., 2016; Mazurek et al., 2017).

Yoshiyuki et al. (2016) studied the effects of sildenafil on right ventricular function in MCT-induced PH rats, and assessed the alteration of right ventricular function in PH rats using the TAPSE index. The TAPSE value of rats measured in the saline control group was 2.3 mm, which was reduced to 1.4 mm after 6 weeks of treatment with MCT. Abdallah Alzoubi et al. (2013) studied the effect of dehydroepiandrosterone (DHEA) on RV of severe PH rats, and they found that TAPSE value of rats in the normal group was 3.45 mm, while TAPSE value in the model group was 2.18 mm, which was significantly lower than that of the normal group.

In addition, Rosas et al. (2023) constructed MCT-induced PH rat models, and measured the mean value of TAPSE to be 3.33 mm in the control group and 1.47 mm in the PH group. Spyropoulos et al. (2020) verified some echocardiographic parameters in the chronic hypoxia and SuHx-induced PH rat models and determined their standardized values. They found that in the chronic hypoxia-induced PH rat model, the mean TAPSE values of the control group and the experimental group were 3.2 mm and 2.1 mm, respectively. In the SuHx-induced PH rat model, the TAPSE values were 2.4 mm and 1.6 mm in the control and experimental groups, respectively. Zhu et al. (2019) established the SuHx-induced PH rat model and the MCT-induced PH rat model, and measured the TAPSE in the A4C view. It was found that TAPSE of the PH group was significantly reduced, and the TAPSE of the control and experimental groups in the SuHx rat model was 2.8 mm and 2.2 mm, respectively. In MCT-induced PH rat model, TAPSE was 3.0 mm and 1.9 mm in the control and experimental groups, respectively.

In humans, TAPSE <17 mm is highly suggestive of right ventricular systolic dysfunction (Zaidi et al., 2020). In PH rat models, there is not study or guideline that gives a clear range of reference values. It may be due to the small size of the rat heart, the complex right ventricular geometrical structure, and much higher heart rate than that of humans that makes ultrasound imaging difficult, *etc.*

#### 5.1.2 Right ventricular systolic excursion velocity (S′)

S' is similar to the TAPSE index and is an important measure of right ventricular longitudinal systolic function. It is a reproducible and easily acquired measure of right ventricular systolic function. S′ is measured by placing the sampled volume in the lateral annulus of the tricuspid valve or the basal segment of the right ventricular free wall in the A4C view and using tissue Doppler to measure right ventricular systolic excursion velocity in this target region (DiLorenzo et al., 2015).

Rosas et al. (2023) constructed the MCT-induced PH rat model. The mean value of S′ was measured to be 62.6 mm/s in the control group and 25.9 mm/s in the PH group. In humans, especially in younger adult patients, S' < 90 mm/s is suspected for abnormal right ventricular function (Zaidi et al., 2020; Lang et al., 2015). In PH rat, there is still without clear range of reference values.

#### 5.1.3 Right ventricular fractional area change (RVFAC)

RVFAC % = (end-diastolic area − end-systolic area) ÷ end-diastolic area × 100%. FAC can be measured by tracing the right ventricular endocardial area in the A4C view. It reflects right ventricular systolic function (Bao et al., 2022; Li et al., 2016).

Rosas et al. (2023) constructed the MCT-induced PH rat model. The mean value of RVFAC was measured to be 44% in control rats and 15% in PH rats. In addition, Zhu et al. (2019) established SuHx-induced PH rat model and MCT-induced PH rat model, measured RVFAC, and found that RVFAC was significantly lower in PH group. In the SuHx-induced rat model, FAC was 72% and 38% in the control and PH groups, respectively; in the MCT-induced PH rat model, FAC was 71% and 52% in the control and PH groups, respectively. In humans, RVFAC are more clinically valuable than right ventricular ejection fraction (RVEF), and the normal reference thresholds for FAC is ≥30% in men and ≥35% in women. When FAC is less than 35%, it is highly suggestive of right ventricular dysfunction (Kou et al., 2014). In PH rat models, there is still without clear range of reference thresholds.

#### 5.1.4 Peak tricuspid flow velocity of the early rapid filling wave (E)/early diastolic myocardial relaxation velocity (E′)

E/E′ is one of the indexes to assess diastolic function of the right heart in rat models. E is the peak tricuspid flow velocity of the early rapid filling wave measured by pulsed Doppler at the tricuspid valve in the A4C view; E′ is the early diastolic myocardial relaxation velocity measured by tissue Doppler in the lateral annulus of the tricuspid valve in the A4C view (Augustine et al., 2018; Cordina et al., 2019).

Wallentin, Egemnazarov et al. (2015) found that the right ventricular E/E′ ratio correlates well with right ventricular pressure. E/E′ allows a reasonably estimation of mean right ventricular pressure values and assessment of right ventricular diastolic function. Larger E/E′ values indicate poorer right heart diastolic function. Rosas et al. (2023) established the MCT-induced PH rat model. The mean value of E/E′ was measured to be 9.79 in the control group and 23.79 in the PH group (P < 0.05). To date, right ventricular diastolic function in the rat has not been adequately studied due to anatomical complexity and technical limitations. As with indexes of systolic function such as TAPSE, there are not studies or guidelines that give a clear range of reference values for E/E′ in rat of PH (Salerno et al., 2025; Schnelle et al., 2018; Wang et al., 2015; Mukherjee et al., 2025).

#### 5.1.5 Right ventricular free wall thickness (RVFWT)

RVFWT is an important index of RVH and reflects the degree of right ventricular wall hypertrophy. It measures the thickness of the right ventricular free wall in the A4C view during diastole using the M-mode or 2D imaging. The measurement is affected by the morphology of RV and the position of the probe, and could not fully reflect the overall structural changes of RV (Alerhand and Adrian, 2023).

Zhu et al. (2019), Kuang et al. (2023) showed that RVFWT measured by ultrasound matched the right ventricular hypertrophy parameter (Fulton’s index) in the SuHx and MCT-induced PH rat models. Recently, Spyropoulos et al. (2020) studied to validate some echocardiographic parameters and determine their standard diagnostic thresholds in hypoxia, MCT, SuHx-induced PH rat models. They found that RVFWT measured by ultrasound could be used as a reliable measure of RV morphology, RVFWT ≥1.03 mm was the diagnostic threshold for RV hypertrophy in PH rat models.

#### 5.1.6 Pulmonary acceleration time (PAT)/pulmonary ejection time (PET)

PAT/PET is the index for assessing PA pressure. PAT is the measurement of the time interval from the onset of pulmonary artery systole to the onset of peak flow velocity using pulsed Doppler in PSAX view. PET is the measurement of the time interval from the onset of right ventricular ejection to the end of ejection. The continuous wave Doppler cursor is aligned with the direction of blood flow during measurement. The accuracy of PAT and PET is influenced by a number of factors such as heart rate, right ventricular function, pulmonary artery compliance and measurement technique (Cordina et al., 2019).

In human echocardiography, PA pressure is assessed by the flow velocities of pulmonary artery and tricuspid regurgitations (Augustine et al., 2018; Parasuraman et al., 2016). Typically, however, these regurgitant flows are not found in the rat model, so PAT/PET are often used as an alternative to assess PA pressure in PH rat.

Zhu et al. (2019) established the SuHx and MCT-induced PH rat model, and found that PAT/PET was significantly decreased in the PH group. In the SuHx-induced PH rat model, the PAT/PET values were 0.44 and 0.22 in the control and PH groups, respectively; in the MCT-induced PH rat model, the PAT/PET values were 0.47 and 0.20 in the control and PH groups, respectively. In addition, Spyropoulos et al. (2020) found that PAT/PET measured by ultrasound can be used as a reliable measure of pulmonary hemodynamics. PAT/PET measured by ultrasound was inversely correlated with RVSP measured by RHC, and PAT/PET ≤0.25 was the diagnostic threshold for PH in rat.

#### 5.1.7 Index of right ventricle-pulmonary artery (RV-PA) coupling: tricuspid annular plane systolic excursion (TAPSE)/pulmonary acceleration time (PAT)

“Right ventricle-pulmonary artery coupling” refers to the interaction between right ventricular contractility and right ventricular afterload. Under physiologic conditions, the two are dynamically regulated to maintain an optimal coupling. However, in pathological conditions, such as PH, right ventricular remodeling and pulmonary vascular remodeling occur, resulting in RV-PA uncoupling. Notably, the RV-PA uncoupling is not an irreversible state, and normal right ventricular-pulmonary artery coupling can be restored by lowering pulmonary artery pressure, or by reducing right ventricular afterload (Vonk Noordegraaf et al., 2017; Sa et al., 2019; Sandeep et al., 2024). The assessment of RV-PA coupling requires the detection of two important indicators, right ventricular systolic function and right ventricular afterload. Examination methods include RHC, ultrasound imaging, and CMR etc (Dong et al., 2022; Bellofiore and Chesler, 2013; Kubba et al., 2016; Aubert et al., 2018).

The ratio of ventricular elasticity [ventricular end-systolic elastance (Ees)] and the ventricular afterload parameter [effective arterial elastance (Ea)] measured by RHC using the pressure-volume catheter (Millar), i.e., the Ees/Ea ratio, is the “gold standard” for assessing RV-PA coupling (Bellofiore et al., 1985; Sanz et al., 2012; Lin et al., 2016). However, RHC is an invasive examination method with the risk of complications such as infection, vascular injury, and pneumothorax, and has limitations in basic research applications. In addition, CMR is noninvasive, but cannot measure ventricular pressure and pulmonary artery pressure directly.

TAPSE/PAT measured by ultrasound integrates the assessment of right ventricular systolic function and right ventricular afterload, and is regarded as a noninvasive surrogate for Ees/Ea ratio evaluation method (Tello et al., 2019; Rako et al., 2023). Yang et al. (2020) established the hypoxia-induced PH rat model and measured that the mean value of TAPSE/PAT was 0.07 mm/s in the control group and 0.06 mm/s in the PH group (P<0.05), suggesting that the RV-PA coupling was altered under hypoxic conditions. In the rat model, there are little studies on TAPSE/PAT index, and there is currently not standardized reference threshold.

### 5.2 Emerging ultrasound imaging techniques in the assessment of cardiac function in PH rats

#### 5.2.1 Speckle tracking imaging (STI)

STI is a technique based on the stability and uniqueness of the echo speckles of myocardial tissue to obtain the strain, strain rate, and motion trajectory of the myocardium by tracking the echo speckles frame by frame. Unlike tissue Doppler, STI is not limited by the direction of the acoustic beam and can analyze myocardial strain in multiple dimensions (longitudinal, circumferential, and radial). Moreover, STI can also track the movement trajectory of myocardial tissue during systole and diastole in real time, and quantify the local myocardial strain through block matching algorithms (e.g., diamond search) to reflect the segmental myocardial function (Kovács et al., 2015; Ming et al., 2013; Salerno et al., 2025; Zacchigna et al., 2021; Bernardo et al., 2017).

**FIGURE 4:**
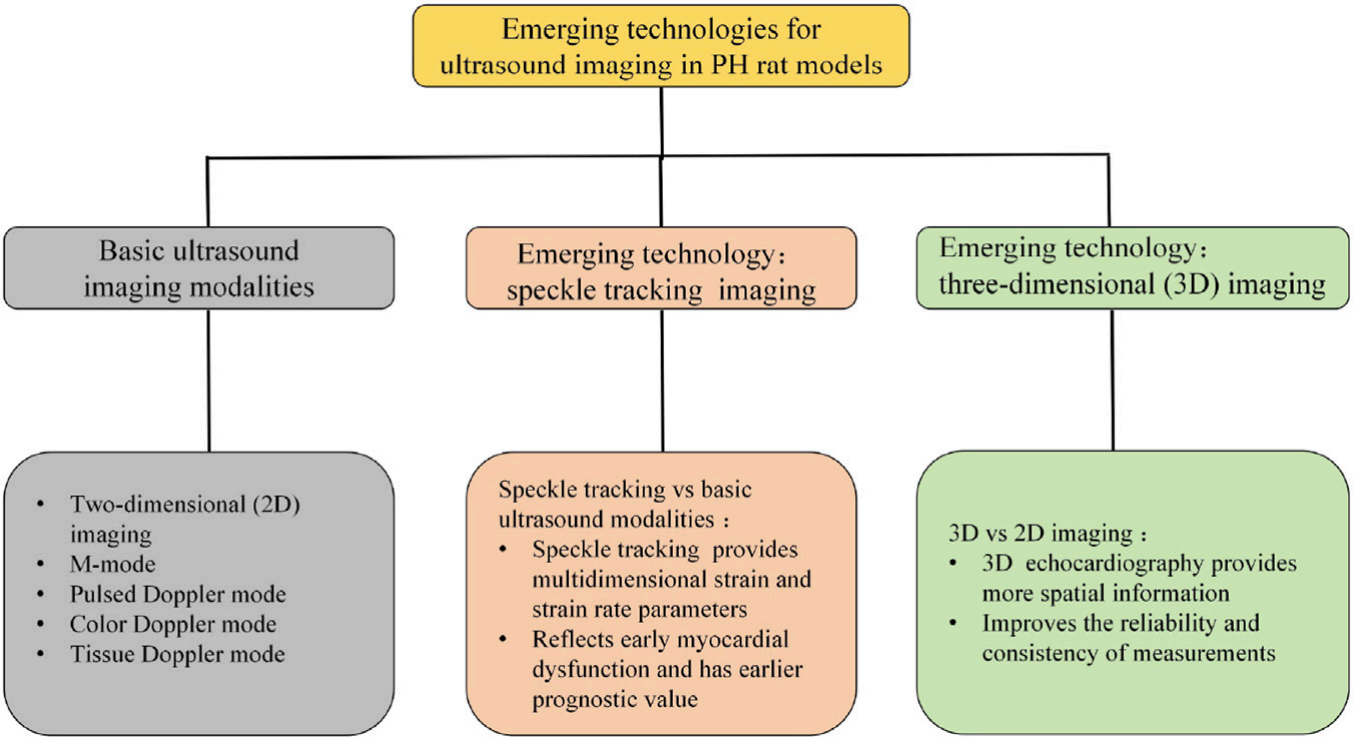
Emerging technologies for ultrasound imaging in PH rat models: speckle tracking imaging and three-dimensional (3D) imaging. Basic ultrasound imaging modalities: two-dimensional (2D) imaging, M-mode, color Doppler mode, pulsed Doppler mode, Tissue Doppler mode. Speckle tracking imaging: speckle tracking vs. basic ultrasound modalities: speckle tracking technology provides multidimensional strain and strain rate parameters, reflects early myocardial dysfunction and has earlier predictive value. 3D imaging: 3D ultrasound imaging vs. 2D ultrasound imaging: 3D echocardiography provides more spatial information, improves the reliability and consistency of measurements.

Clinical studies indicate that STI can reflect early ventricular dysfunction, with predictive value preceding other indicators such as ejection fraction (EF) (Moceri et al., 2022). Rats typically exhibit rapid heart rates (often >300 beats/min), leading to blurred STI images. Ming et al. (2013) maintained a frame rate of 180–220 frames/s during imaging to enhance image clarity and accurately delineate endocardial borders. Additionally, artifacts frequently occur during image acquisition, necessitating synchronous gating with respiratory motion. Anesthesia depth (typically 1%–2% inhaled isoflurane) must balance heart rate stability with physiological state (Kovács et al., 2015; Ming et al., 2013; Salerno et al., 2025). Despite these technical challenges, this technique holds significant potential for early assessment of right ventricular function in disease and is anticipated to become a crucial imaging tool in future echocardiographic studies evaluating pulmonary hypertension (Figure 4).

#### 5.2.2 Three-dimensional (3D) ultrasound

Three-dimensional ultrasound is an examination method that utilizes three-dimensional imaging technology to display cardiac structure and function (Sa et al., 2019; Bellofiore et al., 1985). Building upon two-dimensional ultrasound, this technique acquires ultrasound images in three-dimensional space. A computer system then performs spatial localization, pixel interpolation, and three-dimensional reconstruction to generate a three-dimensional stereoscopic image of the heart. By providing a three-dimensional stereoscopic view of the heart, it enables more accurate assessment of parameters such as cardiac chamber volume, ventricular function, and valve structure, serving as a vital tool for cardiovascular disease research (Rako et al., 2023; Zacchigna et al., 2021). This technique typically employs 1%–2% isoflurane inhalation anesthesia. For rats, probes with frequencies ≥20 MHz are commonly used, operating at frame rates of 600–800 fps to perform real-time 3D volumetric scanning (Yuchi et al., 2022; Tidholm et al., 2024; Grune et al., 2018; Stegmann et al., 2019). Koskenvuo et al. (2010) employed a 30 MHz center frequency transducer coupled to the Vevo®3100 imaging system for three-dimensional image acquisition. This method directly displays the three-dimensional morphology of cardiac chambers without requiring any geometric assumptions about ventricular cavities and remains unaffected by the irregular geometry of diseased chambers. Consequently, it offers greater accuracy than two-dimensional ultrasound measurements and is more advantageous for evaluating ventricular structural and functional characteristics (Figure 4).

## 6 Comparative analysis of ultrasound imaging in the evaluation of the right heart in PH rats

### 6.1 Comparative analysis of right heart ultrasound indexes in three PH rat models

Rosas et al. (2023) established MCT-induced PH rat model by subcutaneous injection of MCT 60 mg/kg in SD rats, and carried out ultrasound detection on the 23rd day. It was observed that this PH model had a significant decrease in TAPSE, suggesting right ventricular systolic dysfunction; an increase in RVFWT, which indicated right heart hypertrophy; a significant decrease in PAT, and a significant decrease in PAT/PET, reflecting pulmonary artery pressure was significantly elevated.

Spyropoulos et al. (2020) exposed SD rats to 9% O_2_ for 2 weeks. Ultrasonographic examination after 2 weeks found that, in these models, TAPSE decreased gradually over time, reflecting gradual deterioration of right ventricular function; RVFWT was significantly increased; PAT and PAT/PET was significantly decreased, reflecting a significant increase in pulmonary artery pressure. Zhu et al. (2019) injected 20 mg/kg Sugen5416 subcutaneously into SD rats, exposed to 10% O_2_ for 3 weeks, and then restored normoxia for 4 weeks. Ultrasonography revealed a significant decrease in TAPSE, reflecting deterioration of right ventricular function; a significant increase in RVFWT, reflecting right ventricular hypertrophy; a significant decrease in PAT and PAT/PET, reflecting a significant increase in pulmonary artery pressure.

In the MCT, chronic hypoxia, and **SuHx**-induced PH rat models, the trend of changes in TAPSE, RVFWT, PAT, and PAT/PET indexes detected by ultrasound is the same, but the degree of change may vary. For example, in the MCT-induced PH model, the TAPSE values were 3.3 mm and 1.4 mm in the control and PH group, respectively, whereas in the SuHx-induced PH model, the TAPSE values were 2.7 mm and 2.0 mm in the control and PH group, respectively. Apparently, the decrease in TAPSE values was more significant in the MCT-induced PH rat model, i.e., the deterioration of right ventricular systolic function was greater in the MCT-induced PH rat model. It suggests that the effect of MCT on the systolic function of right ventricle in the rats is more significant compared to SuHx. In addition, in the chronic hypoxia-induced PH model, the PAT/PET values were 0.3 and 0.2 in the control and PH groups, respectively, whereas in the SuHx-induced PH model, the PAT/PET values were 0.4 and 0.2 in the control and PH groups, respectively. Obviously, the decrease in PAT/PET values was more pronounced in the SuHx-induced PH model, that is, the degree of elevation of pulmonary arterial pressure values was greater. It indicates that SuHx have a more significant effect on elevating pulmonary artery pressure compared to chronic hypoxia (Table 3).

**TABLE 3:** Comparative analysis of right heart echocardiographic indexes in three PH rat models.

Model types	Researcher [references] molding conditions	TAPSE, mm (control vs. model)	RVFWT, mm (control vs. model)	PAT, ms (control vs. model)	PAT/PET
The MCT- induced PH rat model	Rosas et al. (2023) SD rats were injected subcutaneously with MCT 60 mg/kg and detected by ultrasound on day 23	Decline significantly (3.33vs1.47)	Increase significantly (0.59vs1.38)	Decline significantly (32.56vs20.23)	Decline significantly (0.46vs0.27)
The chronic hypoxia-induced PH rat model	Spyropoulos et al. (2020) SD rats were exposed to 9% O_2_ for 2 weeks, and ultrasound detection was performed after 2 weeks	Decline significantly (3.2vs2.2)	Increase significantly (0.7vs1.2)	Decline (22.5vs19.5)	Decline (0.3vs0.2)
The SuHx- induced PH rat model	Zhu et al. (2019) SD rats were injected subcutaneously with Sugen5416 (20 mg/kg), exposed to 10% O_2_ for 3 weeks, then restored to normoxia for 4 weeks, and detected by ultrasound	Decline (2.7vs2.0)	Increase (0.6vs1.5)	Decline significantly (30.3vs17.5)	Decline significantly (0.4vs0.2)

Abbreviations: SuHx, Sugen5416/hypoxia; MCT, monocrotaline; SD, sprague dawley.

### 6.2 Correlation analysis of right heart ultrasound measurements with the gold standard in the PH rat model

#### 6.2.1 Correlation analysis of pulmonary artery pressure indexes

Spyropoulos et al. (2020) showed that PAT/PET values estimated by ultrasound correlated well with PASP measured by RHC. The correlation coefficient between PAT/PET and PASP measured by RHC was 0.787. Zhu et al. (2019) established hypoxia-induced and MCT-induced PH rat model, and found that PAT and PAT/PET estimated by ultrasound correlated well with PASP measured by RHC. The correlation coefficients between PAT and PASP measured by RHC were 0.435, and the correlation coefficient between PAT/PET and PASP measured by RHC were 0.503. In addition, Kuang et al. (2023) established SuHx-induced PH rat model, and the correlation coefficient between PAT estimated by ultrasound and mPASP measured by RHC were 0.768, and that between PAT/PET and mPASP measured by RHC was 0.82. Koskenvuo et al. (2010) established MCT-induced PH rat model, in this model, the correlation coefficient between PAT estimated by ultrasound and mPAP measured by RHC was 0.74, which was also a good correlation (Table 4).

**TABLE 4:** Correlation coefficients between Echo indices and RHC and Fulton’s index.

Correlation coefficients	PAT/PET measured by Echo	RVFWT measured by Echo
PASP measured by RHC	0.787 (Spyropoulos et al., 2020); 0.503 (Zhu et al., 2019); 0.82 (Kuang et al., 2023)	—
Fulton’s index	—	0.491 (Spyropoulos et al., 2020); 0.774 (Zhu et al., 2019)

Abbreviation: Echo, Echocardiography.

#### 6.2.2 Correlation analysis of the right heart structural parameters

It was shown that RVFWT measured by ultrasound was significantly correlated with right ventricular hypertrophy parameters (Fulton’s index). Spyropoulos et al. (2020) constructed the SuHx and the MCT-induced PH rat model. The significant increase in RVFWT measured by ultrasound was matched to Fulton’s index, and the correlation coefficient between RVFWT and Fulton’s index (right ventricular weight/left ventricular and septal weight) was 0.491. Zhu et al. (2019) constructed chronic hypoxia and MCT-induced PH rat model, RVFWT were significantly increased, the correlation coefficient between RVFWT measured by ultrasound and Fulton’s index was 0.774 (Table 4).

### 6.3 Comparison of speckle tracking and basic ultrasound in the assessment of RV in PH

Speckle tracking technology is able to analyze myocardial motion trajectories, provide multidimensional strain and strain rate parameters, and provide a more comprehensive assessment of myocardial functional status (Cameli et al., 2017; Lu et al., 2023). Studies have shown that right ventricular myocardial longitudinal strain (RVLS), circumferential strain (RVCS), and radial strain (RVRS) measured using STI and the corresponding strain rates can be very sensitive to detect early myocardial dysfunction and have earlier predictive value in PH (Kuang et al., 2023; Bernardo et al., 2017; Sachdev et al., 2011). Whereas basic ultrasound parameters such as TAPSE and S′ are also widely used to assess right ventricular function, these parameters usually show significant changes only after a certain degree of disease progression. Therefore, STI is able to detect early myocardial dysfunction more sensitively than basic ultrasound parameters, significantly improve the sensitivity and prognostic predictive power of cardiac function assessment. STI will have significant potential in the assessment of right ventricular function.

## 7 Challenges and future directions

### 7.1 Limitations of right ventricular ultrasound detection in PH rat

Although ultrasound imaging has significant advantages in the assessment of cardiac function in PH rats, there are still some shortcomings, such as differences in technical parameters, non-uniformity of operation specifications, and difficulty in the assessment of RV. Different ultrasound techniques, such as Doppler ultrasound, and speckle tracking, each have their own unique imaging principles and parameter settings. Differences in the image acquisition, processing and analysis processes of these technologies make it difficult to directly compare and integrate results between different technologies.

In addition, the rat right ventricle has an irregular geometry and small size. This complexity of the right ventricular structure results in significant field of view limitations during ultrasound imaging, making it difficult to clearly visualize all regions in one plane (Bernardo et al., 2017). In addition, the presence of right ventricular chordae tendineae and papillary muscles complicates endocardial tracking and measurement, affecting the reproducibility and accuracy of measurements. In addition, RV of the rats is located behind the sternum, and ultrasound propagation is impeded during the detection process, which also increases the difficulty of imaging and leads to a decrease in image quality. Moreover, when the rats have cardiac arrhythmia, the measurement results of ultrasound detection are also affected, reducing the accuracy of measurement (Sanada et al., 2021; Bernardo et al., 2017).

Although some studies have proposed how right ventricular parameters can be measured more accurately and conveniently, such as the adjustment of ultrasound probe position to acquire clear and complete right ventricular images and the use of standard anesthesia methods to ensure that echocardiograms are acquired with similar heart rates in rats, these methods lack a standardized procedure and have not been consistently applied in different studies (Rosas et al., 2023; Bernardo et al., 2017). In addition, the ultrasound equipment and technical parameters (e.g., frequency, resolution, *etc.*) used in different studies may differ, further affecting the reproducibility and consistency of the measurement results.

### 7.2 Future direction: application of multimodal fusion (ultrasound and CMR or micro-CT or AI)

Multimodal fusion (e.g., ultrasound and CMR or micro-CT or AI) has significant potential to overcome the limitations of single imaging technique and achieve complementary advantages, providing a more comprehensive and accurate assessment of cardiac function in PH rats (Ouyang et al., 2024).

Although CMR is time-consuming and costly, it provides information with high spatial, temporal resolution and accuracy, making it the “gold standard” for assessing right ventricular structure and function (Kramer et al., 2020). CMR can be used to assess early myocardial mechanical and functional changes in RV by tissue tracking (TT) (Dong et al., 2024; Bourfiss et al., 2021). Yan and Zhuangzhi (2017) employed CMR for dynamic assessment of right ventricular function, revealing a strong negative correlation between CMR-measured RVEF and mean pulmonary artery pressure (mPAP) obtained *via* right heart catheterization (r = −0.823). Additionally, CMR can quantify right ventricular remodeling reversal following drug therapy (e.g., Rho kinase inhibitors) or interventions (Prisco et al., 2021). 7.0T CMR sensitively detects morphological and functional alterations in pulmonary arteries and the right heart during the progression of PAH in rats, establishing a foundation for investigating the evolutionary mechanisms of PAH (Yuan et al., 2019).

In addition, Micro-CT is uniquely suited for imaging evaluation of fine structures. Micro-CT can obtain high-resolution 3D images in a short period of time to assess the morphology of RV and overall ventricular systolic indexes such as stroke volume and EF (Baktybek et al., 2018; Zelt and Mielniczuk, 2018). By fusing images from different modalities of ultrasound and MRI or micro-CT, the advantages of each can be synthesized to improve the overall imaging resolution and accuracy. For example, ultrasound-guided CMR scans ensure accurate localization, and the high-resolution images provided by CMR can compensate for ultrasound’s lack of detail.

It is also worth noting that in recent years, AI technology has shown great potential in medical image analysis. It can play an important role in multimodal image fusion, for example, it can further improve imaging efficiency and diagnostic accuracy through automatic segmentation, quantitative analysis and data integration. In addition, AI technology can reduce the need for manual labeling and improve the consistency and reproducibility of measurements (Qin et al., 2024; Shi et al., 2022).

**FIGURE 5:**
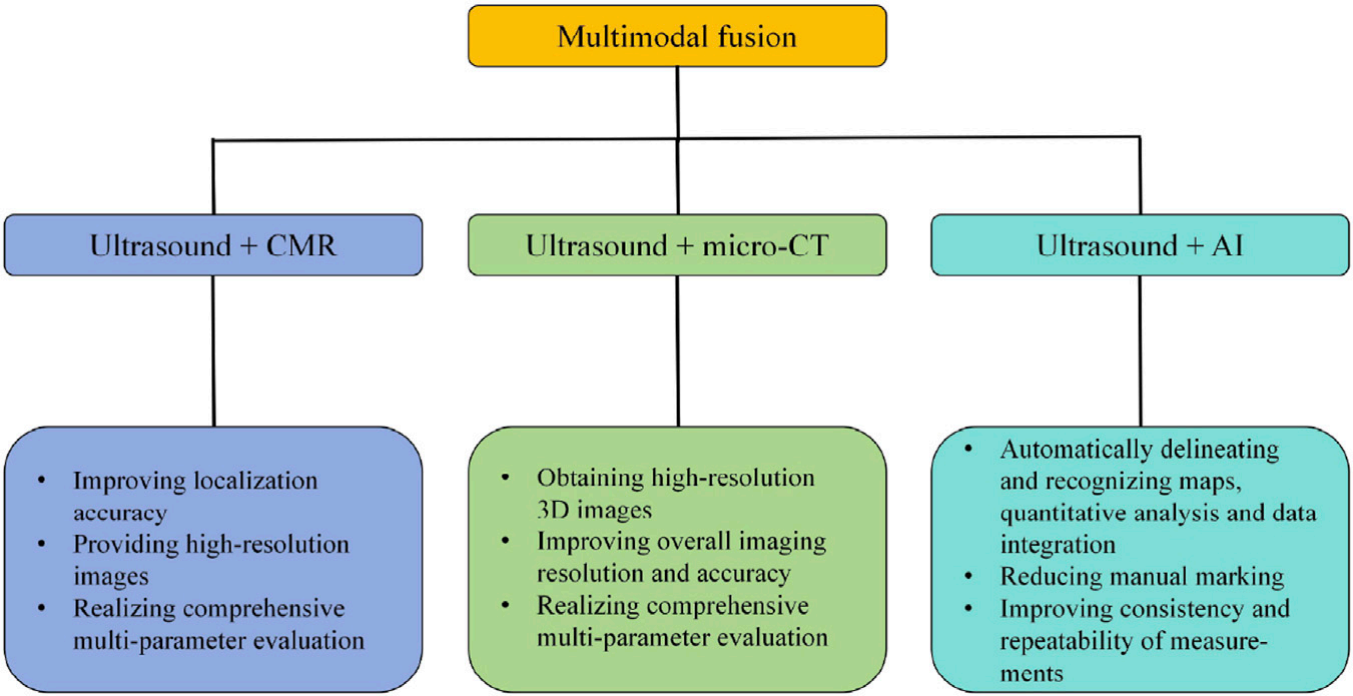
Advantages of multimodal fusion (ultrasound + MRI or micro-CT or AI). Ultrasound + CMR: improving localization accuracy, providing high-resolution images, realizing comprehensive multi-parameter evaluation. Ultrasound + micro-CT: obtaining high-resolution 3D images, improving overall imaging resolution and accuracy, and realizing comprehensive multi-parameter evaluation. Ultrasound + AI: automatically delineating and recognizing maps, quantitative analysis and data integration, reducing manual marking, improving consistency and repeatability of measurements. CMR, Cardiac magnetic resonance; micro-CT, Micro computed tomography; AI, Artificial intelligence.

Multimodal fusion is greatly useful for detection of right ventricular structure and dysfunction, especially in the early stages of PH. And multimodal fusion can realize comprehensive multi-parameter assessment, and better monitoring of progression of PH in the rat models. It will have great potential for future application (Figure 5).

However, multimodal fusion also has certain limitations, such as high costs, availability of high-end imaging equipment, and consistency issues. The acquisition, maintenance, and operational costs of high-end imaging equipment (such as MRI and micro-CT) are extremely high, requiring dedicated laboratory environments (Seo and Lee, 2018). The development and training of artificial intelligence models also necessitate additional computational resources and data annotation labor, further increasing overall costs (Rivera et al., 2024). Many research institutions lack specialized high-resolution imaging equipment, particularly in the field of small animal imaging. This results in poor technological accessibility, necessitating cross-institutional collaboration to achieve multimodal data acquisition. Furthermore, Parameters measured across modalities may exhibit systematic differences, demanding cross-modal calibration (Seo and Lee, 2018). Moreover, AI models require extensive training with high-quality annotated data. However, multimodal imaging data for rats is scarce, and the significant anatomical variability among small animals can easily lead to insufficient model generalization capabilities (Rivera et al., 2024). Therefore, future efforts should focus on enhancing feasibility through standardized protocols, low-cost imaging technologies, and optimized AI algorithms.

## 8 Conclusion

Patients with PH have severe symptoms and extremely poor prognosis, which may eventually lead to RV failure or even death. The evaluation of right heart function is crucial in research on the molecular mechanisms and drug screening of PH. Ultrasound is a non-invasive, radiation-free, real-time examination method, and new technology STI in ultrasound can detect myocardial dysfunction earlier and more sensitively, and 3D ultrasound generates 3D stereo images of the heart, which can more accurately assess the morphology and functional changes of RV, and is well suited for the dynamic monitoring of the heart in the PH rat model. Commonly used ultrasound indexes to assess the structure and function of the right heart in PH rat include TAPSE, RVFAC, RVFWT, PAT/PET *etc.* Due to the complex structure of the right heart and fast heart rate in rats, currently only RVFWT and PAT/PET have been established clear diagnostic thresholds in studies on reference thresholds for these indexes. RVFWT ≥1.03 mm is the threshold for right heart hypertrophy and PAT/PET ≤0.25 is the threshold for the diagnosis of PH in rat. And reference thresholds for other indexes are to be studied. In addition, correlation analysis of ultrasound indexes with the gold standard was done and found that PAT, PAT/PET measured by ultrasound with PASP measured by RHC; PAT measured by ultrasound with mPAP by RHC; and RVFWT measured by ultrasound with Fulton’s index, showed good correlation.

**FIGURE 6:**
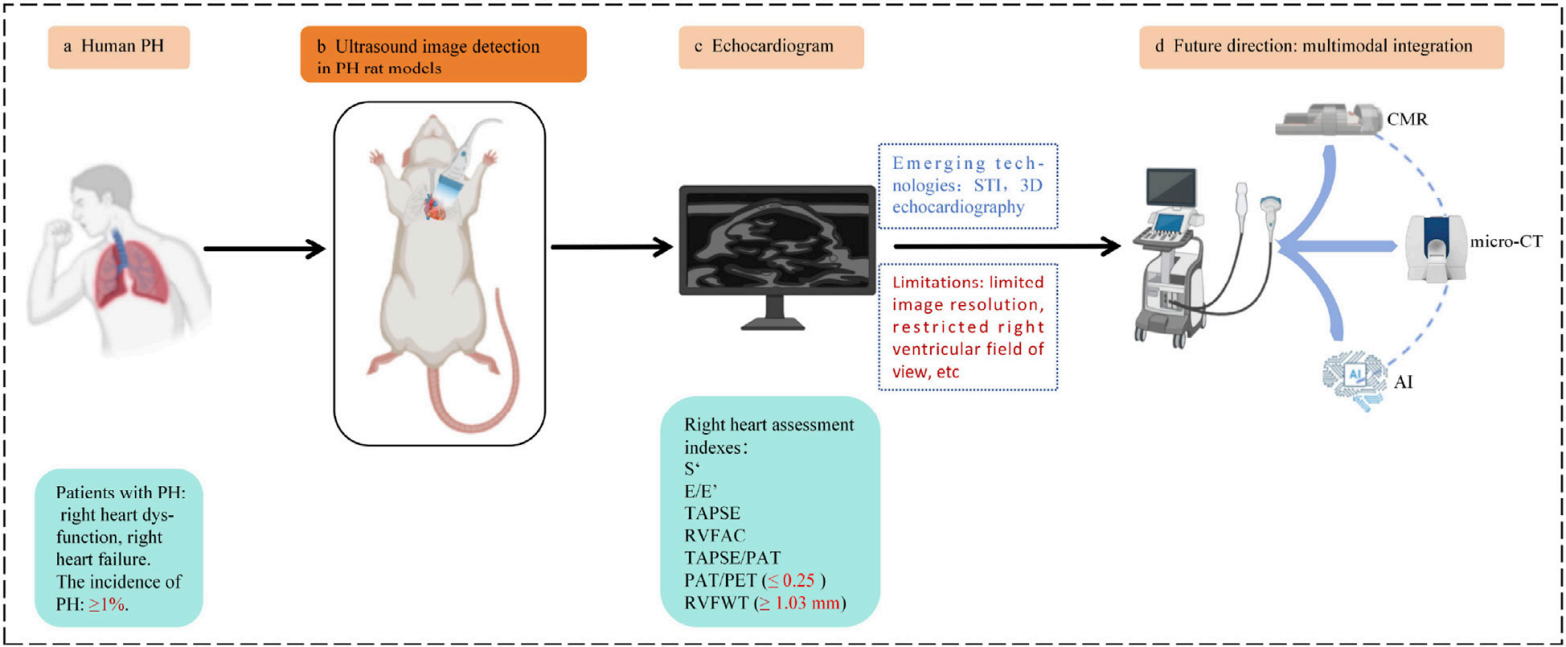
**(a)** Patient with PH: right heart dysfunction, RV failure. The incidence of PH: ≥1%. **(b)** Ultrasound image detection in the PH rat models. **(c)** Echocardiogram. Right heart assessment indexes: PAT/PET ≤0.25 is the diagnostic threshold for PH in rat, RVFWT ≥1.03 mm is the diagnostic threshold for RVH in PH rat. **(d)** Future direction: Multimodal integration: ultrasound + MRI or micro-CT or AI. Original figure created with BioRender.com. S’, Right ventricular systolic excursion velocity; E/E’, Peak tricuspid flow velocity of the early rapid filling wave/early diastolic myocardial relaxation velocity; TAPSE, Tricuspid annular plane systolic excursion; RVFAC, Right ventricular fractional area change; TAPSE/PAT, Tricuspid annular plane systolic excursion/pulmonary acceleration time; PAT/PET, Pulmonary acceleration time/pulmonary ejection time; RVFWT, Right ventricular free wall thickness; RV, Right ventricular hypertrophy; PH, Pulmonary hypertension; STI, Speckle tracking imaging; 3D echocardiography, Three-dimensional echocardiography; CMR, Cardiac magnetic resonance; micro-CT, Micro computed tomography; AI, Artificial intelligence.

However, ultrasound imaging also has its limitations, such as the lack of standardized operation procedures, limited image resolution, restricted right ventricular field of view, and difficulty in imaging the right heart. The multimodal fusion of ultrasound and MRI or micro-CT or AI could comprehensively utilize the advantages of each, improve the overall imaging resolution and accuracy, and realize the multi-parameter comprehensive assessment, which might be the future development trend and direction of cardiac function assessment in PH rat (Figure 6). Accurate assessment of right ventricular function in PH rats lays an important foundation for research into the molecular mechanisms and drug screening of PH.

## Glossary

TAPSE: Tricuspid annular plane systolic excursion
RVFAC: Right ventricular fractional area change
RVFWT: Right ventricular free wall thickness
PAT: Pulmonary acceleration time
PET: Pulmonary ejection time
RHC: Right heart catheterization
CMR: Cardiac magnetic resonance
micro-CT: Micro computed tomography
AI: Artificial intelligence
PH: Pulmonary hypertension
mPAP: Mean pulmonary artery pressure
PAH: Pulmonary arterial hypertension
MCT: Monocrotaline
RV: Right ventricular
RVH: Right ventricular hypertrophy
RVF: Right ventricular failure
CO: Cardiac output
SuHx: Sugen5416/hypoxia
PVR: Pulmonary vascular resistance
SD: Sprague dawley
CHD: Congenital heart disease
PWD: Pulsed wave doppler
FS: Fractional shortening
TDI: Tissue Doppler imaging
PLAX: Parasternal long-axis
PSAX: Parasternal short-axis
A4C: Apical four-chamber
DHEA: Dehydroepiandrosterone
RVEF: Right ventricular ejection fraction
RV-PA: Right ventricle-pulmonary artery
PASP: Pulmonary artery systolic pressure
STI: Speckle tracking imaging
EF: Ejection fraction
RVLS: Right ventricular myocardial longitudinal strain
RVCS: Right ventricular circumferential strain
RVRS: Right ventricular and radial strain
TT: Tissue tracking
